# *In Vivo* Labelling of Adenovirus DNA Identifies Chromatin Anchoring and Biphasic Genome Replication

**DOI:** 10.1128/JVI.00795-18

**Published:** 2018-08-29

**Authors:** Tetsuro Komatsu, Charlotte Quentin-Froignant, Irene Carlon-Andres, Floriane Lagadec, Fabienne Rayne, Jessica Ragues, Ralph H. Kehlenbach, Wenli Zhang, Anja Ehrhardt, Kerstin Bystricky, Renaud Morin, Jean-Michel Lagarde, Franck Gallardo, Harald Wodrich

**Affiliations:** a CNRS UMR 5234, Microbiologie Fondamentale et Pathogénicité, Université de Bordeaux, Bordeaux, France; b NeoVirTech SAS, ITAV, Université de Toulouse, CNRS, Toulouse, France; c Department of Molecular Biology, Faculty of Medicine, Göttingen Center of Biosciences (GZMB), Georg-August-University Göttingen, Göttingen, Germany; d Chair for Virology and Microbiology, Witten/Herdecke University, Witten, Germany; e LBME, Centre de Biologie Intégrative, CBI, Université de Toulouse, CNRS, Toulouse, France; f IMACTIV3D SAS, ITAV, Université de Toulouse, CNRS, Toulouse, France; International Centre for Genetic Engineering and Biotechnology

**Keywords:** DNA tagging, adenovirus, chromatin association, in vivo labelling, incoming genome, live cell imaging, nuclear transport, viral replication

## Abstract

Adenoviruses are DNA viruses with a lytic infection cycle. Following the fate of incoming as well as recently replicated genomes during infections is a challenge. In this study, we used the ANCHOR3 technology based on a bacterial partitioning system to establish a versatile *in vivo* imaging system for adenoviral genomes. The system allows the visualization of both individual incoming and newly replicated genomes in real time in living cells. We demonstrate that incoming adenoviral genomes are attached to condensed cellular chromatin during mitosis, facilitating the equal distribution of viral genomes in daughter cells after cell division. We show that the formation of replication centers occurs in conjunction with *in vivo* genome replication and determine replication rates. Visualization of adenoviral DNA revealed that adenoviruses exhibit two kinetically distinct phases of genome replication. Low-level replication occurred during early replication, while high-level replication was associated with late replication phases. The transition between these phases occurred concomitantly with morphological changes of viral replication compartments and with the appearance of virus-induced postreplication (ViPR) bodies, identified by the nucleolar protein Mybbp1A. Taken together, our real-time genome imaging system revealed hitherto uncharacterized features of adenoviral genomes *in vivo*. The system is able to identify novel spatiotemporal aspects of the adenovirus life cycle and is potentially transferable to other viral systems with a double-stranded DNA phase.

**IMPORTANCE** Viruses must deliver their genomes to host cells to ensure replication and propagation. Characterizing the fate of viral genomes is crucial to understand the viral life cycle and the fate of virus-derived vector tools. Here, we integrated the ANCHOR3 system, an *in vivo* DNA-tagging technology, into the adenoviral genome for real-time genome detection. ANCHOR3 tagging permitted the *in vivo* visualization of incoming genomes at the onset of infection and of replicated genomes at late phases of infection. Using this system, we show viral genome attachment to condensed host chromosomes during mitosis, identifying this mechanism as a mode of cell-to-cell transfer. We characterize the spatiotemporal organization of adenovirus replication and identify two kinetically distinct phases of viral genome replication. The ANCHOR3 system is the first technique that allows the continuous visualization of adenoviral genomes during the entire virus life cycle, opening the way for further in-depth study.

## Abstract (precis)

Viruses must deliver their genomes to host cells to ensure replication and propagation. Characterizing the fate of viral genomes is crucial to understand the viral life cycle and the fate of virus-derived vector tools. Here, we integrated the ANCHOR3 system, an *in vivo* DNA-tagging technology, into the adenoviral genome for real-time genome detection. ANCHOR3 tagging permitted the *in vivo* visualization of incoming genomes at the onset of infection and of replicated genomes at late phases of infection. Using this system, we show viral genome attachment to condensed host chromosomes during mitosis, identifying this mechanism as a mode of cell-to-cell transfer. We characterize the spatiotemporal organization of adenovirus replication and identify two kinetically distinct phases of viral genome replication. The ANCHOR3 system is the first technique that allows the continuous visualization of adenoviral genomes during the entire virus life cycle, opening the way for further in-depth study.

## INTRODUCTION

Upon infection, viruses need to rapidly initiate viral gene expression to enlarge their repertoire of actions to counteract host antiviral mechanisms and to take control of cellular functions of the host for viral propagation. Adenoviruses (AdV) are nonenveloped, nuclear-replicating DNA viruses, which in most cells start a lytic replication cycle within a few hours of nuclear genome delivery. The ∼36-kb linear double-stranded DNA (dsDNA) of the adenoviral genome is arranged in viral chromatin, termed the core, inside the virion (1, 2). The genome itself is organized into compacted, irregularly spaced nucleoprotein complexes through several hundred copies of the histone-like core protein VII and charge-neutralizing core proteins V and X (1–3). Two copies of the terminal protein (TP) are covalently attached to the 5′ end of the AdV genome. To deliver their genomes, AdV enter cells by receptor-mediated endocytosis (4, 5), escape from the endosomal compartment in an autophagy-assisted process by engaging with motor proteins, and are transported to the nucleus (5–11). This delivery strategy is very efficient and shields the genome inside the capsid from cytosolic DNA sensors. Genomes are released from capsids at the nuclear pore complex (NPC) and imported through the NPC into the nucleus (12, 13). The imported genome entity retains TP and protein VII, while protein V is lost prior to import (14, 15). Protein VII is the only protein that can be clearly detected upon nuclear transport of viral genomes (16), driving genome import by accessing nuclear import receptors (17, 18) and protecting the genome from the DNA damage response (19, 20). Very little is known about the fate of newly imported genomes, due to the lack of adequate tools to detect single viral genomes. Because protein VII is abundantly bound to the viral genome, antibodies detecting protein VII label viral genomes at least in the first hours of infection (21–24), although loss of protein VII from viral genomes over time has not been studied in detail. Incoming genomes are thought to be retained inside the nucleus by attachment to the nuclear matrix through the covalently bound TP, but the nature of this attachment remains elusive (25, 26). Depending on the cell type, AdV genomes start replicating within 8 to 12 h postinfection (hpi), and late gene expression, encoding structural proteins, is initiated by the major late promoter in a replication-dependent manner (27). Replication of AdV DNA occurs through a single-stranded intermediate and coincides with the formation of replication centers (RC), which are morphologically identifiable by the single-stranded DNA (ssDNA) binding protein (DBP) or cellular marker proteins, such as the ubiquitin-specific protease USP7 (28–30). Newly synthesized dsDNA accumulates at the peripheries of RC and spreads into the surrounding nucleoplasm, where transcription occurs (29, 31). We recently showed two morphologically distinct RC, suggesting that early RC produce genomes associated with cellular histones, adapted for late gene expression, while late RC may produce transcriptionally silenced genomes devoid of cellular histones for virion packaging. Late replicated genomes subsequently accumulate in a specific nuclear area, which we termed virus-induced postreplication (ViPR) bodies (32). ViPR bodies are surrounded by late RC and labeled by the nucleolar protein Mybbp1A (32). Subsequent genome assembly and packaging of progeny virions are thought to occur at the peripheries of RC, largely based on the observation of the accumulation of assembly components and intermediates *in situ* (33).

Direct detection of AdV genomes has been a technological challenge to studying AdV morphogenesis. Fluorescence *in situ* hybridization (FISH) has been used to detect both incoming and replicated AdV genomes (13, 34, 35), but the harsh sample preparation processing destroys the morphological context. Metabolic labeling of viral genomes is another recently developed technique for detecting incoming single viral genomes, as well as replicated viral DNA in cells (32, 36–39). For this approach, viruses are replicated in cells supplemented with chemically modified nucleoside analogs, such as EdU (5-ethynyl-2′-deoxyuridine) and EdC (5-ethynyl-2′-deoxycytidine). Inside the producer cell or following virion purification and *de novo* infection, individual genomes can be visualized using click chemistry under mild conditions compatible with antibody detection. Applied to AdV, this approach confirmed that most imported genomes are bound by protein VII (36) and permitted the identification of early versus late RC (32). While metabolic labeling provides great spatial resolution, temporal resolution is limited to pulse-chase applications that do not permit *in vivo* observation. Early attempts to genetically label AdV genomes for *in vivo* imaging used multiple copies of the *tet* operator, replacing the E1 region and E1-complementing cells expressing green fluorescent protein (GFP)-tagged *tet* repressor. This system allowed labeling of capsid-associated genomes from incoming particles in living cells in real time but failed to detect genomes at later stages of infection, e.g., upon or after nuclear import (34). We recently used a different strategy to visualize intranuclear genomes. Immediate-early adenoviral gene expression (E1A) occurs within hours of infection and requires conversion of viral genomes from their condensed transport form to a transcriptionally active configuration (24). The cellular acidic protein TAF-Iβ/SET associates with AdV genomes through interaction with protein VII (40) immediately upon nuclear entry (22, 41) and is necessary for rapid E1A gene expression, suggesting a role for TAF-Iβ in initial viral chromatin unpacking (22, 42, 43). We exploited the TAF-Iβ–protein VII association and showed that cell lines expressing GFP-tagged TAF-Iβ form spots in the nucleus, depicting single incoming genomes in living cells (41). Using this first functional *in vivo* imaging system for individual intranuclear AdV chromatin complexes, we showed that AdV avoids recognition by most known nuclear DNA sensors and prevents transcriptional silencing (39, 44, 45). Despite its functionality, the system requires genome-bound protein VII, and its removal, e.g., upon replication, limits *in vivo* observations to the early infection phase.

The ANCHOR3/ParB system is an *in vivo* DNA-tagging system that was shown to minimally affect DNA metabolism and has been successfully applied to study dsDNA break repair and single-gene transcription in living cells in real time (46, 47). The system is derived from the bacterial partitioning system ParB-*parS*, which activates mitotic segregation in bacteria (48). The underlying principle of DNA tagging with the ANCHOR3/ParB system is the oligomerization of a fluorescently labeled OR3/ParB protein with a short (>1-kb) specific dsDNA seed sequence containing several *parS* sites, resulting in fluorescent spots at *parS* sites *in situ*. In this study, we adopted the ANCHOR3 system to tag AdV genomes. We showed that ANCHOR3 identifies individual incoming genomes, as well as newly replicated genomes, in living cells in real time. Using this system, we demonstrated that AdV genomes are associated with cellular chromatin during cell division. Using specific markers for early and late RC, we further showed that AdV genome replication occurs in two kinetically distinct phases associated with a switch in RC morphology. Our data showed low-level early replication. In contrast, late replication was associated with strong genome amplification and coincided with ViPR body formation. Altogether, we present a novel, versatile AdV genome-tagging strategy that allowed us for the first time to follow individual incoming genomes over time with the simultaneous spatiotemporal analysis of RC formation and replicated DNA.

## RESULTS

### *In vivo* detection of incoming AdV genomes using ANCHOR3 technology.

**FIG 1:**
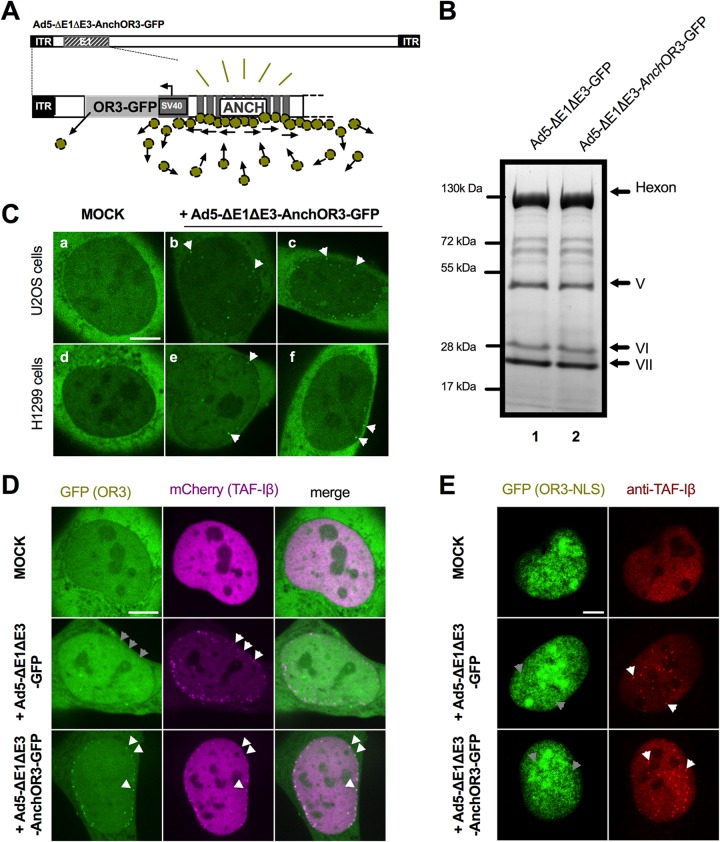
Detection of incoming AdV genomes through direct DNA labeling. (A) Schematic overview of ANCHOR3 encoding AdV vector with E1/E3 deleted. The OR3-GFP expression cassette (light gray) is upstream of the *Anch* sequence consisting of 10 nucleation seeds for binding and oligomerization of the OR3-GFP protein (green spheres). (B) Coomassie gel analysis of purified virus particles without (lane 1) or with (lane 2) inserted ANCHOR3 sequence. Major capsid proteins are indicated on the right. (C) U2OS (top row) or H1299 (bottom row) epithelial cells were transfected with OR3-GFP expression plasmid and either mock infected (a and d) or infected with Ad5-ΔE1ΔE3-ANCHOR3-GFP (b,c,e, and f). At 3 hpi, cells were imaged by spinning-disk microscopy, and individual frames are shown. OR3-GFP spots representing viral genomes are indicated by arrows (see Movie S1A to C in the supplemental material). (D) Stable mCherry-TAF-Iβ-expressing U2OS cells were transfected with OR3-GFP expression plasmid and either mock infected (top row), infected with Ad5-ΔE1ΔE3-GFP (middle row), or infected with Ad5-ΔE1ΔE3-ANCHOR3-GFP (bottom row). At 3 hpi, the cells were imaged by spinning-disk microscopy, and individual frames are shown. An overlay of the GFP signal (left column) and the mCherry signal (middle column) is shown in the right column. Individual genomes are indicated by white arrows and their hypothetical positions by gray arrows. (E) As in panel D using OR3-GFP-transfected U2OS cells. Cells were fixed at 3 hpi and stained with anti-TAF-Iβ antibodies. The GFP signal (green) and the TAF-Iβ signal (red) are shown, and individual genomes and their hypothetical positions are indicated by white and gray arrows, respectively. Scale bars, 10 μm.

The ANCHOR3 system is derived from the bacterial partitioning complex and was originally developed to directly tag cellular DNA and to visualize and measure DNA processing in real time in living cells (46, 49, 50). To adapt the system to visualize incoming and newly replicated adenoviral genomes, we incorporated the ANCHOR3 system into the E1 region of a HAdV-C5-derived vector with E1/E3 deleted. The inserted ∼3.5-kb sequence contained an expression cassette for the OR3 protein fused to the N terminus of GFP and placed upstream of the “*Anch*” cassette comprising a sequence of 10 nucleation sites for OR3-GFP binding and oligomerization (46) (Fig. 1A). The newly generated vector (Ad5-ΔE1ΔE3-ANCHOR3-GFP) was amplified in E1-complementing HEK293 cells. The vector production yield was 4.8 × 10^9^ physical particles (pp)/μl at 4.6 × 10^7^ PFU/μl, both comparable to the yield and infectivity obtained for a conventional AdV type 5 (Ad5)-GFP-expressing vector with E1/E3 deleted (Ad5-ΔE1ΔE3-GFP). Cells in individual plaques showed a distinct nuclear GFP stain (data not shown), and the particle composition of vectors containing the ANCHOR3 system was indistinguishable from that of normal vectors, suggesting that the ANCHOR3 system did not affect virus growth and/or capsid composition (Fig. 1B). The principle of the ANCHOR3 system is that the OR3-GFP protein binds to the *Anch* sequence, which induces localized protein oligomerization, resulting in the appearance of a fluorescent spot *in situ*. We thus wanted to know if, upon infection, *Anch*-tagged viral genomes could be detected. We transfected cells with an OR3-GFP expression vector, because expression from the viral genome is delayed upon initial infection. Noninfected control cells showed a homogeneous, predominantly cytoplasmic distribution of the OR3-GFP protein irrespective of the cell line used (Fig. 1C) (data not shown). Cells infected with Ad5-ΔE1ΔE3-ANCHOR3-GFP displayed individual fluorescent spots appearing within 1 to 3 hpi inside the nuclei of living cells (Fig. 1C), timing that coincides with genome delivery kinetics (41). Using transfected cells expressing large or small amounts of either OR3-GFP or a nuclear version of OR3 (OR3-NLS-GFP) confirmed that the spots were infection dependent and restricted to the nucleus (data not shown).

To confirm that the fluorescent spots were indeed viral genomes, and to determine whether all incoming genomes would result in spot formation, we used the alternative detection method of labeling AdV genomes with fluorescent TAF-Iβ (41). TAF-Iβ is immediately recruited to imported AdV genomes by binding to genome-associated protein VII upon nuclear entry (41). We coexpressed mCherry-tagged TAF-Iβ and OR3-GFP (Fig. 1D) and infected the cells with viruses containing genomes with or without *Anch* sequence. Mock-infected cells did not display fluorescent spots (Fig. 1D; see Movie S1A in the supplemental material). In contrast, we observed fluorescent nuclear TAF-Iβ spots upon nuclear import, which were negative for OR3-GFP in the absence of the *Anch* sequence and positive for OR3-GFP in the presence of the *Anch* sequence (Fig. 1D; see Movie S1B and C in the supplemental material). Interestingly, in the latter case, almost all genomes were double positive. Because both labels are independently recruited to the viral genome, this suggested that (i) the ANCHOR3 system was very efficient in labeling all incoming genomes and (ii) within the first 3 hpi, all the genomes retained at least some protein VII. To investigate this association further, we fixed infected OR3-GFP-expressing cells at 3 hpi and stained either for genome-bound protein VII or for endogenous TAF-Iβ. Incoming genomes could be specifically labeled with anti-protein VII antibodies (data not shown) or with TAF-Iβ antibodies in the presence or absence of the ANCHOR3 sequence (Fig. 1E). In contrast, ANCHOR3-containing genomes did not produce fluorescent spots (Fig. 1E) irrespective of the fixation protocol, suggesting that individual incoming viral genomes marked by OR3-GFP cannot be fixed (see Discussion).

**FIG 2:**
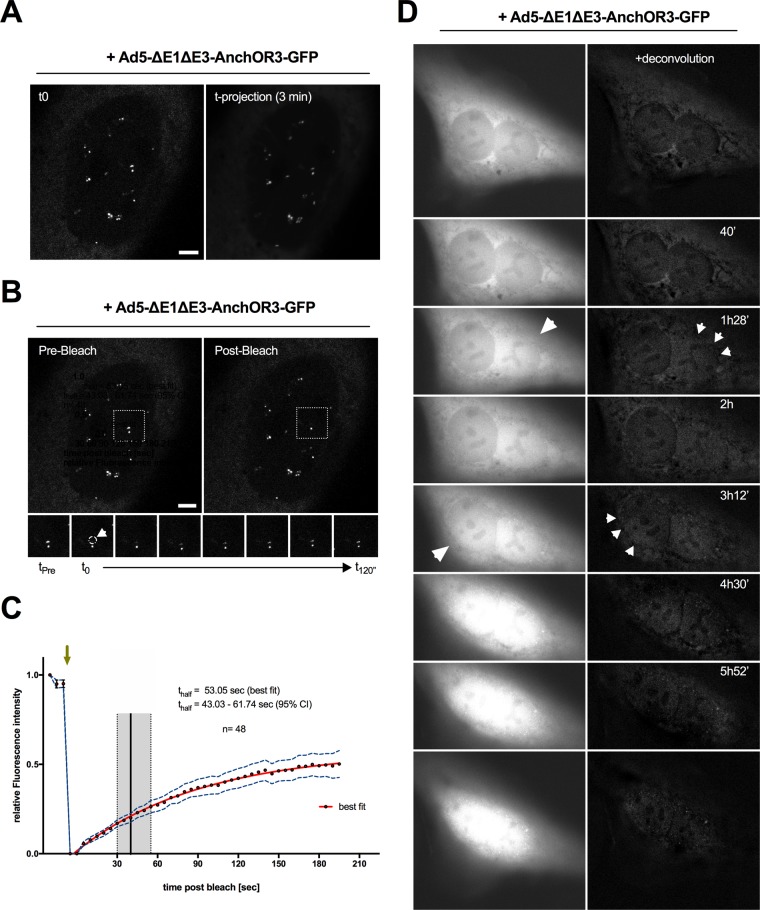
Dynamic analysis of incoming intranuclear genomes. (A) At 24 hpi, intranuclear OR3-GFP- labeled genomes were imaged by time-lapse confocal microscopy at 1 frame/3 s. A single frame is depicted on the left and a projection over 3 min depicting absence of genome mobility on the right. (B) FRAP analysis of the same nucleus as in panel A showing a single genome before (left) and after (right) bleaching. Individual frames on the bottom are from the boxed region showing OR3-GFP fluorescence recovery on the bleached genome. (A and B) Scale bars, 5 μm. (C) FRAP recovery curves of individual genomes. The green arrow indicates the time of photobleaching application, and the *x* axis indicates time postbleaching. The red line indicates the best fit of the recovery curve with the 95% CI (dotted blue line; *n* = 48). The half-recovery time is indicated by the black vertical line (best fit) or the gray box (95% CI). (D) OR3-GFP-expressing cells were infected with Ad5-ΔE1ΔE3-ANCHOR3-GFP and imaged at 1 frame/8 min (see Movie S2 in the supplemental material). Time-stamped individual frames are shown as raw images (left) or following deconvolution (right), depicting breakdown of the permeability barrier of the nuclear envelope (large arrows) and intranuclear genomes (small arrows).

Fluorescent OR3-GFP spots in living cells showed confined movement (Fig. 2A), resembling intranuclear dynamics previously shown for AdV genomes using the TAF-Iβ detection system (41). To confirm that GFP signals on genomes showing confined movement occurred through dynamic recruitment of OR3-GFP proteins, we next performed fluorescence recovery after photobleaching (FRAP) analysis on individual genomes. OR3-GFP-expressing cells were infected with Ad5-ΔE1ΔE3-ANCHOR3-GFP. At 24 hpi, nuclei displaying several individual OR3-GFP spots were targeted to bleach individual genomes (Fig. 2B). The half-recovery times were calculated for >40 individual genomes, showing that recovery was fast and occurred within ∼53 s (95% confidence interval [CI], 43.03 to 61.74 s) (Fig. 2C), which is in good agreement with previously determined OR3-GFP recovery kinetics in a heterologous cell system (47). We next performed time-lapse microscopy on OR3-GFP-expressing cells over the first hours of infection with the *Anch* sequence-bearing vector to visualize the kinetics of appearance of fluorescent spots in living cells in real time (Fig. 2D; see Movie S2 in the supplemental material). The analysis revealed that OR3-GFP spots were detectable in the nuclei of infected cells within 1 to 3 hpi, often close to the nuclear envelope. After their appearance, positioning of OR3-GFP spots within the nucleus remained relatively stable (Fig. 2A and D). Interestingly, dot appearance was often preceded by a sudden influx of GFP signal into the nucleoplasm, confirming breakdown of the diffusion barrier of the nuclear envelope (Fig. 2D), which was previously suggested to accompany capsid disassembly at the NPC (51).

### Incoming AdV genomes are associated with cellular chromatin during cell division.

**FIG 3:**
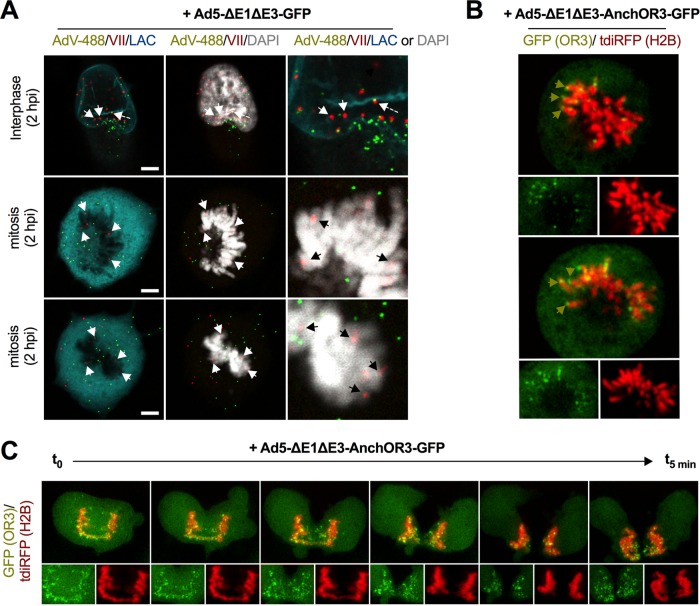
Chromatin anchoring of incoming AdV genomes. (A) Cells were infected with Alexa 488-labeled virus particles (AdV-488; green signal) and fixed at 2 hpi. Viral genomes were stained with protein VII-specific antibodies (VII; red signal), cellular chromatin was stained with DAPI (4′,6-diamidino-2-phenylindole) (gray signal), and the nuclear envelope was stained with lamin A/C-specific antibodies (LAC; cyan signal) as indicated. The top row depicts a cell in interphase, and the middle and bottom rows a cell in mitosis. Scale bars, 5 μm. The white and black arrows indicate chromatin-associated genomes. The dashed arrow indicates a genome still associated with capsid at the nuclear envelope. Note that the images are Z-stack projections. (B) Cells expressing fluorescent histone H2B (tdiRFP; red signal) and OR3-GFP (green signal) were imaged at 3 hpi using live-cell imaging. The large images show mitotic cells using the combined signals; the small images below show separate channels for viral (left; green) and cellular (right; red) chromatin. The green arrows indicate chromatin-associated viral genomes. (C) As in panel B, showing several still images of a live-cell imaging sequence of separating chromosomes with associated viral genomes with ∼1 min between frames (see Movie S3 in the supplemental material).

Early reports suggested that AdV genomes associate with the nuclear matrix (26). Although the nuclear matrix, defined as a distinct biochemical subcellular fraction, has no specific markers for immunostaining, lamin A/C is thought to be one of the components commonly found in nuclear matrix preparations (52). To investigate this association, we fixed cells infected with nonreplicative Alexa 488-labeled Ad5-ΔE1ΔE3-GFP at 2 hpi and stained them for genome-associated protein VII and for lamin A/C to delineate the nuclear envelope (NE). In interphase cells, genomes bound to protein VII could be clearly identified inside the nucleus or at the nuclear rim, whereas virus particles either associated with the nuclear rim or remained outside (Fig. 3A). Occasionally, we observed cells that had entered mitosis, identified by condensed cellular chromatin and a diffuse lamin A/C stain, indicating NE disassembly. Most AdV genomes in mitotic cells associated with condensed cellular chromatin, suggesting a specific interaction (Fig. 3A). We next repeated the experiment using a cell line stably expressing fluorescent histone H2B to label cellular chromatin in living cells and transfected these cells with OR3-GFP expression vectors. Cells were infected with *Anch* sequence encoding AdV and analyzed by live-cell imaging at 3 hpi. Mitotic cells were identified by the presence of condensed fluorescent chromosomes. *Anch*-bearing AdV genomes formed fluorescent spots, which in living cells associated with condensed cellular chromatin (Fig. 3B), similar to protein VII-stained genomes in fixed mitotic cells. When mitotic cells were imaged through mitosis, chromatin-bound AdV genomes were distributed equally between daughter cells (Fig. 3C; see Movie S3 in the supplemental material). Altogether, by using the ANCHOR3 system, we were able to demonstrate that AdV genomes attach to cellular chromatin in living cells, which may facilitate their symmetric distribution in daughter cells.

### *In vivo* detection of replicated AdV genomes using the ANCHOR3 system.

**FIG 4:**
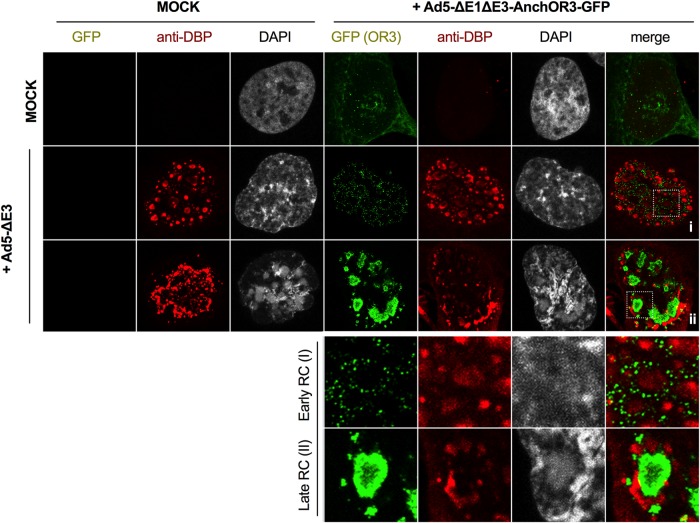
ANCHOR3-GFP in AdV genome replication in fixed cells. Cells were mock infected or infected with Ad5-ΔE1ΔE3-ANCHOR3-GFP in the absence (top [long] row) or presence (middle and bottom [long] rows) of replicative Ad5-ΔE, as indicated on the top and left. At 24 hpi, cells displaying GFP fluorescence (green signal) were fixed and stained with DBP-specific antibodies (red signal). The long middle row shows cells with early RC and the long bottom row cells with late RC as defined previously (32). The two short rows at the bottom show enlargements of the boxed region in the last column depicting replicated DNA in early (I) and late (II) RC, respectively.

**FIG 5:**
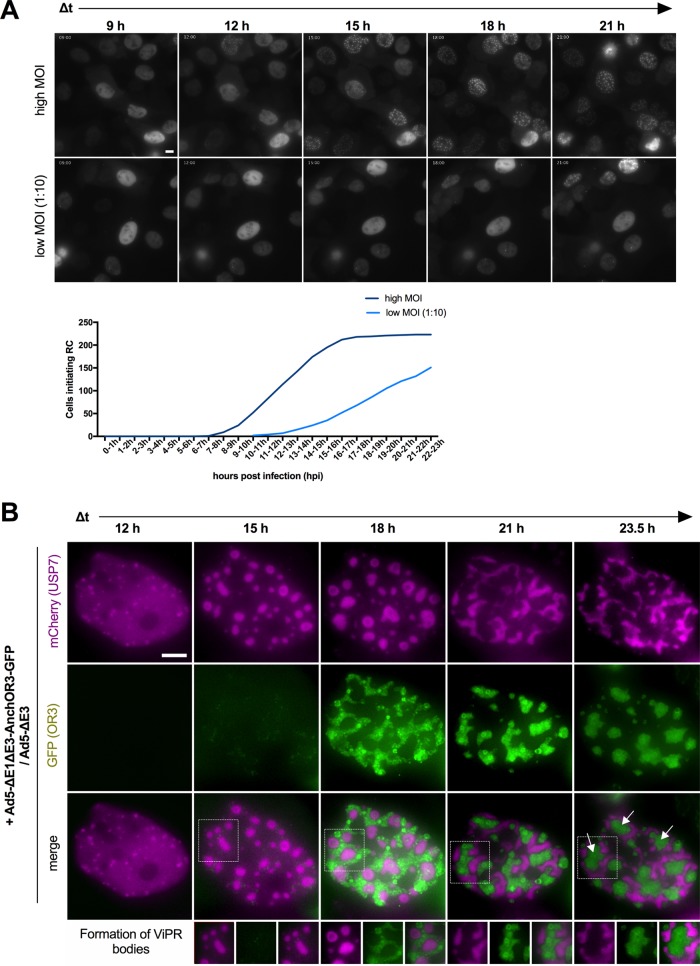
ANCHOR3-GFP in AdV genome replication in living cells. (A) Cells stably expressing mCherry-USP7 display RC upon infection. Representative fields of view are shown at 3-h intervals for high MOI and for lower MOI at 1:10 dilution. The graph shows the absolute number of cells starting replication from 10 independent fields of view. Note that at high MOI, all the cells replicate by ∼15 hpi. (B) Stable mCherry-USP7-expressing cells were infected with an excess of Ad5-ΔE1ΔE3-ANCHOR3-GFP in the presence of replicative Ad5-ΔE3. A representative cell is shown, using individual frames from live-cell imaging at 3-h intervals (12 to 23.5 hpi), as indicated. The spatiotemporal organization of RC (top row; USP7 signal; magenta) and replicated genomes (middle row; OR3-GFP signal; green) and the spatial relationship of both signals (bottom row) are shown (see Movie S4 in the supplemental material). The boxed areas display formation of ViPR bodies, indicated by white arrows in the last panel (see the text for details). Scale bars, 10 μm.

The ANCHOR3 system permitted the detection of incoming viral genomes in living cells. We next asked whether the system could also detect replicated viral genomes. AdV genome replication occurs through a single-stranded intermediate where the ssDNA is bound by the DBP. Because the ANCHOR3 system recognizes only dsDNA, ssDNA can be detected by labeling the DBP, which can be used to label viral RC; according to the literature, replicated dsDNA genomes are expected to accumulate at the peripheries of viral RC (29, 32). We thus infected noncomplementing U2OS cells with replicative (E1-positive) nontagged AdV or with a mixture of replicative nontagged AdV and an excess of nonreplicative Ad5-ΔE1ΔE3-ANCHOR3-GFP vector. In this setup, the replicative virus complements the deleted E1 region of the vector. At 24 hpi, cells receiving the mixture displayed intranuclear GFP-positive structures. Cells were fixed and stained with DBP-specific antibodies to detect viral RC (Fig. 4). Cells that received only replicative virus showed no GFP signal but stained positive for DBP, identifying either “early” or “late” RC, as described previously (30, 32) (Fig. 4). The DBP signal in cells receiving the virus mixture was virtually identical. In addition, these cells showed two different distributions of the GFP signal linked to the morphology of the DBP stain. In cells displaying early RC, replicated genomes accumulated at the periphery and could be distinguished as individual spots (Fig. 4). In contrast, in cells showing late RC, viral genomes were packed into large and dense structures flanked by DBP-positive domains (Fig. 4). The results suggested a close link between the appearances of RC and replicated, GFP-positive viral genomes. This also indicated that replicated AdV genomes can be fixed without losing specific OR3-GFP association, although the same approach failed with incoming AdV genomes (Fig. 1E) (see Discussion). The cellular protein USP7 phenocopies the DBP distribution during the replication cycle (30, 32). We therefore generated cells stably expressing USP7 tagged with mCherry to mark RC formation in living cells in real time. In uninfected cells or prior to the onset of replication, USP7 was homogenously nucleoplasmic (data not shown; see Movie S4 in the supplemental material). Cells with tagged USP7 were infected with replicative, ANCHOR3-negative viruses to provide E1A expression in *trans* and an excess of ANCHOR3-GFP-positive vectors with E1 deleted at two different multiplicities of infection (MOI) and imaged for 24 h at 20-min interval between frames. Using high MOI, cells started to form small USP7 clusters in the nucleoplasm at ∼9 to 15 hpi, showing that the onset of replication occurs in a narrow time window after initial infection (Fig. 5A). At a 10-fold-diluted MOI, USP7 cluster formation was delayed and more spread out, suggesting that the amount of virus inoculum determines the onset of RC formation (Fig. 5A). The appearance of USP7 clusters coincided with the appearance of GFP spots associated with the clusters (Fig. 5B; see Movie S4). USP7 clusters increased in size and formed early RC, while the number of GFP spots also increased and eventually surrounded the RC. Over time, the USP7 signal adopted the form of late RC associated with large densely packed GFP-positive assemblies of replicated genomes (Fig. 5B), forming ViPR bodies (32). In summary, our results showed that the ANCHOR3 system readily identifies replicated AdV genomes and that the system is amenable to tracking the spatiotemporal distribution of AdV genome replication directly in living cells with minimal invasion.

### AdV DNA is replicated in two kinetically different replication phases.

**FIG 6:**
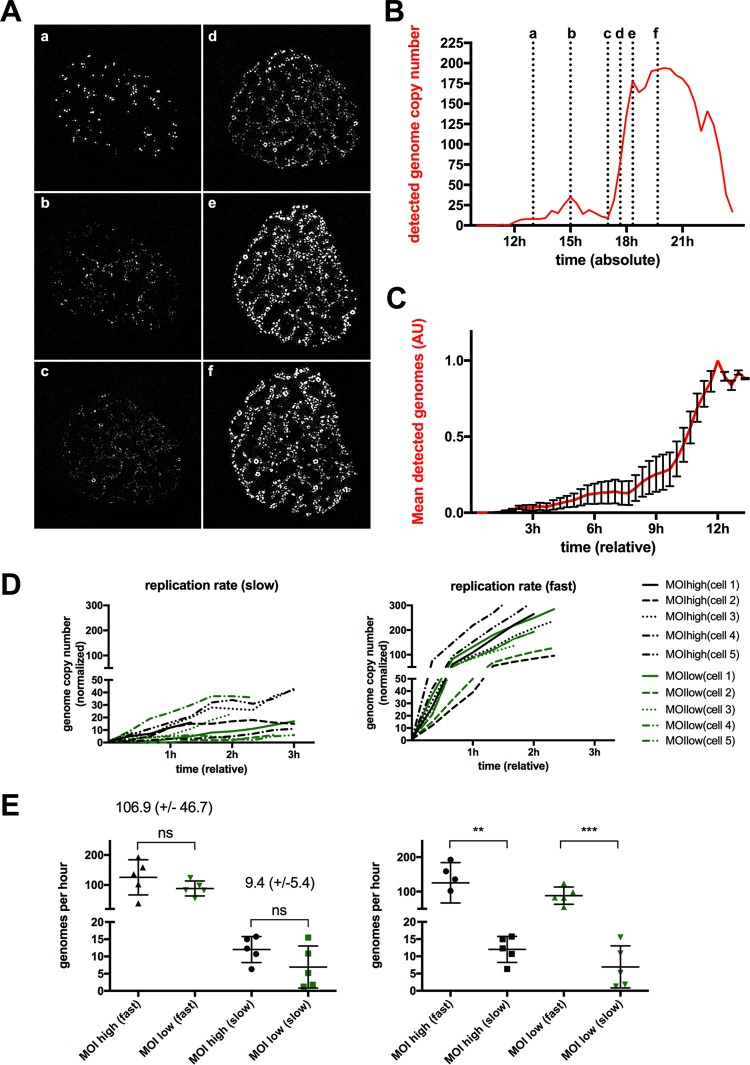
Biphasic AdV genome replication. (A) Stably mCherry-USP7-expressing cells were infected with a mixture of replicative Ad5-ΔE3 with an excess of Ad5-ΔE1ΔE3-ANCHOR3-GFP vector. The images are individual frames showing the development of the OR3-GFP signal during AdV replication. The depicted time points (a to f) are as indicated in panel B. (B) Number of detected genomes measured in individual OR3-GFP signals using a representative cell (shown in panel A) over time. The *x* axis depicts time postinfection. The dotted vertical lines and letters indicate the time points of the respective frame shown in panel A. (C) Mean genome copy numbers estimated from the OR3-GFP signals from 10 cells. The signals were normalized and set to *t*_0_ at the onset of replication. The error bars indicate standard errors of the mean (SEM). (D) Numbers of genome copies present in the OR3-GFP signals during early/slow (left) and late/fast (right) replication phases. The absolute number of genomes and timing were normalized to the same starting point. Slow and fast replication phases for individual cells are depicted on both graphs in the same colors (black for high MOI and green for low MOI, as indicated in the legend). (E) Replication rates from individual cells in panel D were calculated by linear least-squares regression individually for low and high MOI, as well as for slow and fast replication phases. The values are derived from cells infected at high MOI (black) or low MOI (green). The indicated values correspond to the means and standard deviations (SD) for high replication rates and low replication rates (*n* = 10 cells).

Imaging AdV replication *in vivo* suggested that AdV genome replication was coordinated in time and space. Therefore, we analyzed the formation of RC and viral replication in more detail. To generate clean quantifiable signals, we first selected individual representative cells from infections performed at high MOI. We separated the channels and subtracted the background signal (data not shown). The number and the size of individual USP7 clusters and the number of individual OR3-GFP spots were determined frame by frame and plotted over the acquisition time. Figure 6A shows a representative cell undergoing genome replication. Each image corresponds to a defined time indicated in the corresponding quantification of the OR3-GFP signal plotted against time postinfection (Fig. 6B). The number of individual OR3-GFP spots revealed a biphasic replication with an initial phase of low-level replication. The early replication phase corresponded to an increase in cytosolic OR3-GFP accumulation, thus avoiding a detection problem due to insufficient OR3-GFP expression (data not shown). At a certain point, the number of individual OR3-GFP spots increased dramatically, suggesting a major shift in the dsDNA genome formation rate until the signal reached a plateau (Fig. 6B) and collapsed upon cell disintegration. Interestingly, while the signal showed a strong increase in intensity, in this second replication phase, the distribution inside the nucleus remained relatively stable (Fig. 6A and B). The OR3-GFP signal was quantified in 10 individual cells. The normalized data were plotted starting when the first GFP spots became visible to obtain the relative time for each step (Fig. 6C). The analysis confirmed the biphasic replication model. Initial genome amplification remained low for ∼6 to 9 h in all analyzed cells, suggesting that only small amounts of progeny dsDNA were formed in this early phase of the replication cycle. All the cells showed a clear switch to a second phase with much higher amplification rates during a short period lasting ∼1 to 2 h (Fig. 6C). To confirm this observation, we determined relative replication rates by plotting the number and intensity of visible individual genomes during slow and fast replication phases over time for 10 individual cells (Fig. 6D). To estimate the effect of the amount of input virus on genome replication rates, we used five cells with high MOI and 5 cells with a 10-fold-lower MOI for the analysis. The average replication rate during both replication phases was calculated by linear least-squares regression and estimated at 9.4 (±5.4) genomes/h for early “low” replication rates and at 106.9 (±46.7) genomes/h for late “high” replication rates, suggesting more than a 10-fold difference in early versus late genome replication rates (Fig. 6E). In contrast, replication rates did not depend on the amount of input virus (Fig. 6E).

**FIG 7:**
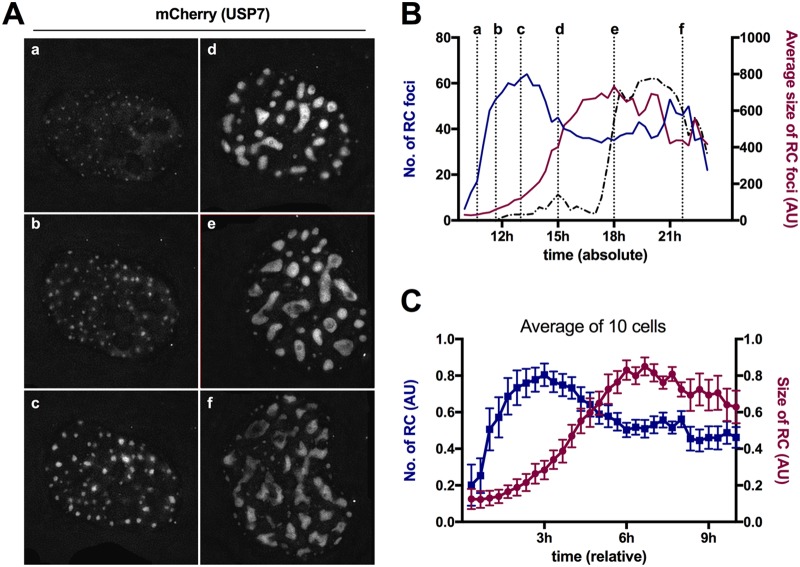
RC dynamics during AdV replication. (A) Stably mCherry-USP7-expressing cells were infected with a mixture of replicative Ad5-ΔE3 with an excess of Ad5-ΔE1ΔE3-ANCHOR3-GFP vector. The images are individual frames showing the mCherry-USP7 signal indicative of the RC during AdV replication. The depicted time points (a to f) are as indicated in panel B. (B) Quantification of the RC number (blue graph) and RC average size (magenta graph) from a representative cell (shown in panel A) over time. The *x* axis depicts time postinfection. The dotted vertical lines and letters indicate the time points of the respective frames shown in panel A. (C) Quantification of RC numbers (blue graph) and RC average sizes (magenta graph) from 10 cells. Signals were normalized and set to *t*_0_ at the onset of RC formation. The error bars indicate SEM.

We next performed a similar analysis for the formation of RC using the mCherry-USP7 signal in the same cell (Fig. 7A). We plotted numbers and sizes of USP7 clusters against time postinfection, showing that clusters formed rapidly to reach a plateau, from which they started to fuse and grow in size until they reached a stable population that fragmented over time (Fig. 7B). We extracted the same information from 10 individual cells. Normalized data were plotted starting at the onset of USP7 cluster formation to obtain the relative time for each step (Fig. 7C). This analysis confirmed that individual USP7 clusters formed rapidly everywhere in the nucleoplasm within ∼3 h to reach a maximum number. For all the cells, we observed that clusters formed suddenly and almost simultaneously without apparent preferred localization except the exclusion of nucleoli. Once the maximum number of clusters was reached, clusters started to fuse and grow in size for an additional ∼3 h until a stable population of early RC was reached. This stable population converted within ∼6 h into late RC, marked through partial fragmentation, depicted as a slight reversal of size and number of USP7 clusters (Fig. 7A to C). Individual GFP spots appeared adjacent to USP7 clusters as soon as they formed (Fig. 5B; see Movie S4 in the supplemental material), suggesting that USP7 clusters are functional RC upon formation. Superimposition of the biphasic DNA replication curve onto USP7 cluster analysis (Fig. 7B) showed that the first genome amplification phase was correlated with the appearance, growth, and fusion of the RC. In contrast, the fast second genome amplification phase correlated with conversion from early into late RC and genome accumulation in their centers.

### Mybbp1A reorganization coincides with rapid genome amplification.

**FIG 8:**
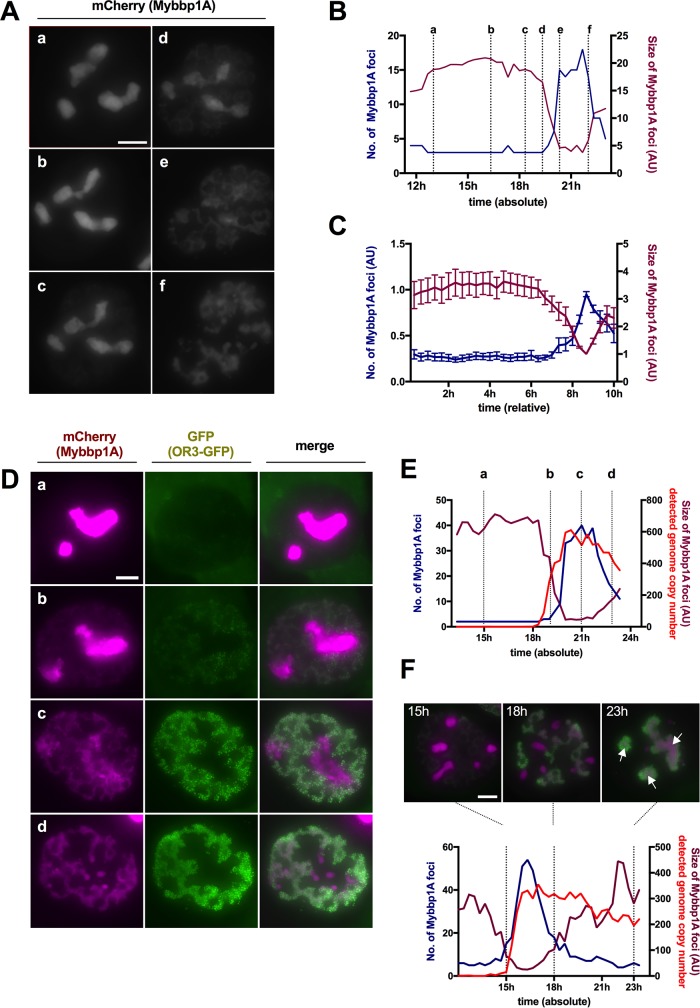
Mybbp1A reorganization during AdV replication. (A) Stably mCherry-Mybbp1A-expressing cells were infected with a mixture of replicative Ad5-ΔE3 with an excess of Ad5-ΔE1ΔE3-ANCHOR3-GFP vector. The images are individual frames showing the mCherry-Mybbp1A signal during AdV replication. The depicted time points (a to f) are as indicated in panel B. (B) Numbers of Mybbp1A foci (blue graph) and average sizes of Mybbp1A foci (magenta graph) from a representative cell (shown in panel A) over time. The *x* axis depicts time postinfection. The dotted vertical lines and letters indicate the time points of the respective frame shown in panel A. (C) Number sof Mybbp1A foci (blue graph) and average sizes of Mybbp1A foci (magenta graph) from 10 cells. The signals were normalized and set to *t*_0_ at the onset of genome replication. The error bars indicate SEM. (D) As in panel A, but a different cell showing the Mybbp1A signal (left column; magenta), the OR3-GFP signal (middle column; green), and their spatial relationship in the overlay (right column). The rows (a to d) depict time points as indicated in panel E. (E) Numbers of Mybbp1A foci (blue graph), their average sizes (magenta graph, normalized to detected genomes), and the numbers of detected genomes measured in individual OR3-GFP signal (red graph) from a representative cell (shown in panel D) over time. The *x* axis depicts time postinfection. The dotted vertical lines and letters indicate the time points of the respective frames shown in panel D. (See Movie S5 in the supplemental material.) (F) Numbers of Mybbp1A foci (blue graph), their average sizes (magenta graph, normalized to detected genomes), and the numbers of detected genomes measured in individual OR3-GFP signals (red graph) as in panel E from a cell in early second replication phase. The *x* axis depicts time postinfection, and the dotted vertical lines indicate the respective frames shown above the graph at 15 hpi, 18 hpi and 23 hpi. Note the concentration of the signal into ViPR bodies late in infection (arrows). Scale bars, 10 μm.

We showed previously through metabolic labeling that replicated AdV genomes have different morphologies during early and late phases of AdV replication (32). In late phases, replicated viral DNA accumulates in ViPR bodies, defined morphologically through association with the nucleolar protein Mybbp1A (32). To investigate if the observed biphasic viral DNA replication could account for the formation of ViPR bodies, we generated stable U2OS cells expressing mCherry-tagged Mybbp1A. Cells were infected as before using a mixture of replicative Ad5-ΔE3 with an excess of Ad5-ΔE1ΔE3-ANCHOR3-GFP vector and imaged for 24 h at 20-min intervals (Fig. 8; see Movie S5 in the supplemental material). Mybbp1A remained in nucleoli until late phases of infection, when it relocalized to replicated viral dsDNA to form ViPR bodies in the nucleoplasm (Fig. 8). We determined the number and sizes of Mybbp1A foci during replication. The analysis of a representative cell (Fig. 8A) showed that Mybbp1A remained associated with nucleoli upon infection for prolonged times (∼20 h under the MOI used), followed by a short period during which Mybbp1A separated from nucleoli and formed heterogeneous structures inside the nucleoplasm (Fig. 8A and B). Quantification of the Mybbp1A signals from 10 different cells and normalization of the signals revealed that this redistribution from the nucleoli to the nucleoplasm occurred ∼6 to 8 h after the onset of viral replication, as determined by the appearance of nuclear OR3-GFP spots (Fig. 8C). We next quantified Mybbp1A redistribution and viral DNA replication in the same cell (Fig. 8D). Again, the first phase of low-level genome replication did not significantly alter the Mybbp1A distribution (Fig. 8E). In contrast, during the second replication phase, marked by a strong increase in genome abundance, Mybbp1A relocated concomitantly with genome amplification until it was completely relocated from nucleoli, suggesting a strong and intricate link between the second phase of genome amplification and Mybbp1A. Mybbp1A associated with replicated genomes formed ViPR bodies, which condensed over time (Fig. 8F), as previously proposed (32).

**FIG 9:**
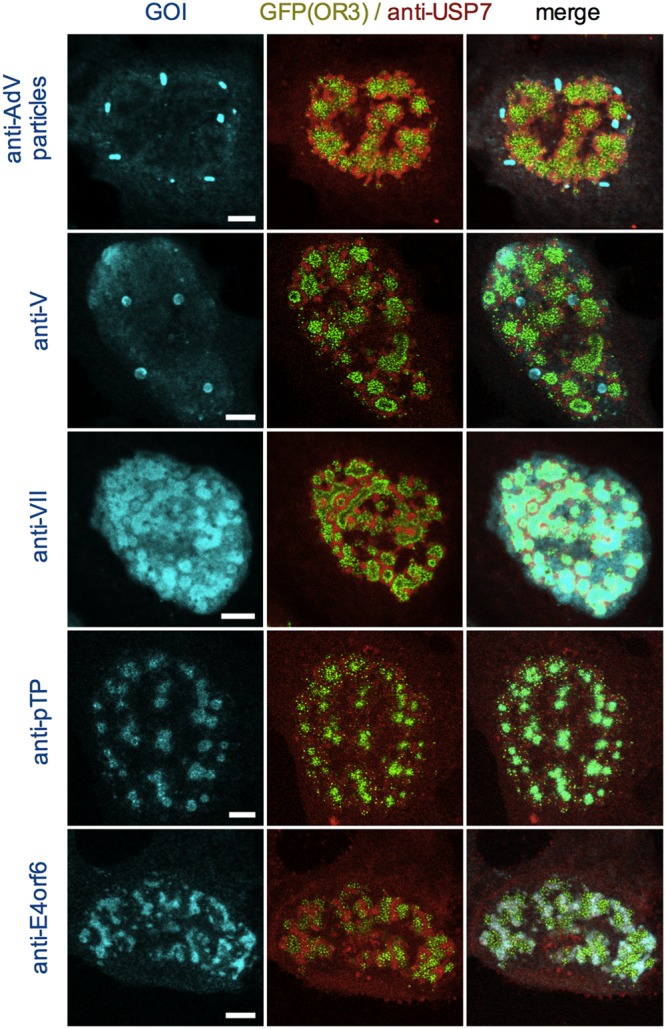
Association of core proteins with ANCHOR3-GFP-tagged genomes. Cells were infected with a mixture of replicative Ad5-ΔE3 with an excess of Ad5-ΔE1ΔE3-ANCHOR3-GFP vector and fixed at 24 hpi. The cells show replicated genomes (green signal) in ViPR bodies surrounded by USP7 detected with specific antibodies (red signal) and costained with antibodies (cyan signal) against AdV particles (top row), protein V (second row), protein VII (third row), pTP (fourth row), and E4orf6 (bottom row), as indicated on the top and left. Scale bars, 5 μm.

One characteristic of genomes accumulating in ViPR bodies is the absence of cellular histones. Here, we identified ViPR bodies with viral genomes through the ANCHOR3 system and by staining cells for endogenous USP7, which surrounds ViPR bodies upon formation (Fig. 9). ViPR bodies were clearly separated from areas stained with whole-particle-derived antibodies, suggesting that particle assembly does not occur in ViPR bodies (Fig. 9). In support of this idea, we never observed OR3-GFP stain associated with virus particles, suggesting that packaged genomes are devoid of bound OR3-GFP, probably due to spatial restriction in the capsid interior. Core protein V was neither excluded nor enriched in ViPR bodies and showed some specificity for nucleoli (53) (Fig. 9), while protein VII was enriched in ViPR bodies, as previously described (32) (Fig. 9), but also stained cellular chromatin (reference 54 and data not shown). TP (and/or its precursor, pTP) was the only viral protein found specifically associated with viral genomes in ViPR bodies, confirming its covalent linkage to replicated genomes (55) (Fig. 9). Interestingly, E4orf6, the recently proposed portal protein for viral genome packaging (56), colocalized with the USP7 signal surrounding ViPR bodies, although a functional link between late replication centers and genome packaging has not been established (Fig. 9). Altogether, our analysis shows that tagging AdV genomes with the ANCHOR3 system provides the necessary means to precisely link the timing of individual replication and genome-processing steps to subnuclear regions marked by the accumulation of viral and/or cellular factors.

## DISCUSSION

The dynamic visualization of viral nucleic acids in living cells is technically challenging but vital to understand the spatiotemporal context and fate of individual viral genomes, virus-derived gene transfer tools, or vaccination vectors. Also, imaging viral genomes throughout the complete viral cycle from infection to viral replication and cell lysis has never been achieved. In this study, we utilized a novel autonomous *in vivo* fluorescent labeling technique called ANCHOR3 to visualize AdV genomes, which we applied to single incoming genomes, as well as to *de novo* replicated genomes. Living cells infected with ANCHOR3 vector showed intranuclear fluorescent spots (Fig. 1), which appeared with kinetics similar to those observed for genome import and only when OR3-GFP was expressed and the *Anch* sequence was present in the vector genome. The simultaneous binding of the observed OR3-GFP spots by fluorescent TAF-Iβ confirmed that these spots were viral genomes, as TAF-Iβ is independently recruited via genome-bound protein VII (41). Most incoming genomes localized close to the nuclear periphery, but we found occasional genomes elsewhere in the nucleoplasm with no apparent preference. We observed fluorescently tagged genomes only inside the nucleus independently of OR3-GFP subcellular localization and/or its expression levels, which is somewhat in contradiction to a recent study suggesting that genome import is a major limitation in infection (36). The authors used metabolically labeled genomes and observed that several capsid-free AdV genomes accumulated in the cytosol, suggesting inefficient nuclear import of AdV genomes. The reason for this discrepancy is unclear but may be related to differences in the MOI used or differences in genome detection or label accessibility (chemical versus genetic modification). For incoming genomes, we were not able to preserve the spot fluorescence upon fixation, which marked individual genomes in living cells. This prevented us from codetecting the OR3-GFP label with protein VII-specific antibodies. In contrast, fixed replicated AdV genomes retained the OR3-GFP association and could be imaged in combination with antibody staining (Fig. 4 and 9). We speculate that the difference could be in part due to different AdV chromatin structures before and after DNA replication (43, 57). Such a difference may affect the dynamic accumulation of OR3 proteins at the *Anch* sequence. Through FRAP analysis, we estimated the half-recovery time for OR3 proteins on incoming viral genomes to be ∼53 s, which is in good agreement with the previously estimated ∼56 s in a cellular model (47).

Live-cell imaging showed that genome import into the nucleus was often accompanied by an increase in the permeability of NE immediately before viral genomes appeared in the nucleus. Our observation supports previous accounts of an increase in NE permeability during AdV genome import (51) and allows us to address the underlying mechanisms in future studies using real-time analysis. Increased permeability of the NE linked to nuclear genome import was also reported for parvoviruses, another nonenveloped DNA virus (58), suggesting that modifications of the NE and/or NPC could be a common genome delivery feature for some DNA viruses.

Dynamic analysis of nuclear AdV genomes in interphase cells using either the OR3 system (Fig. 2) or the TAF-Iβ system (41) suggest restricted mobility, implying specific genome anchoring inside the nucleus. Early literature reports showed that AdV genomes attach to the nuclear matrix defined by biochemical extraction protocols (26). Here, we used microscopy and single-genome detection by protein VII staining to show that AdV genomes associate with cellular chromatin in cells that have entered mitosis after infection. In agreement with this observation, the vast majority of OR3-GFP-labeled genomes were found associated with condensed cellular chromosomes in living cells and were distributed when still tethered to chromosomes between dividing cells (Fig. 3). Such association between cellular chromatin and AdV genomes was proposed decades ago (59, 60) but was not studied in living or dividing cells. We thus showed unequivocally that adenoviral vector genomes use chromatin association to be transferred between dividing cells. Our observation may help to understand and directly address vector persistence *in vivo* in gene therapy or vaccination models, which mostly rely on indirect measures of transgene expression levels or of a biological response of the cell to the transgene. Several binding modes, which may involve cellular histones and other chromatin binding proteins, have been proposed for tethering of viral genomes to chromosomes (61). Future studies will address whether the association with cellular chromatin also plays a role in the confined movement of incoming AdV genomes observed in interphase cells, independently of or coordinately with nuclear matrix interaction.

In our previous study, we provided evidence for the formation of two morphologically distinct RC (early and late RC), as well as the formation of ViPR bodies accumulating replicated AdV genomes delimited by late RC (32). Here, we followed RC formation in living cells in real time by monitoring tagged USP7 as a surrogate marker, which mimics DBP distribution in infected cells. RC formation was imaged together with direct detection of replicated AdV genomes through the ANCHOR3 system, enabling us to directly measure *in vivo* genome replication rates. To our knowledge, this is the first real-time attempt of this kind, and we successfully imaged dynamic formation of RC and amplification of AdV genomes in living cells. The results demonstrate that viral genome amplification occurs in two distinct phases. We observed that RC formation is initiated by a sudden appearance of small dot-like USP7 structures scattered all over the nucleoplasm associated with a few replicated genomes. Reducing the MOI delays initial RC formation, suggesting that a viral factor(s) is involved in the early onset of RC formation. We consider DBP a possible candidate because it forms homo-oligomers through its C terminus in a concentration-dependent manner, assembling RC-like structures even in the absence of a viral infection (44, 57, 62). An attractive and simple model could be that DBP expression levels have to reach a threshold that allows RC formation by generating DBP oligomers as a function of the intranuclear concentration. This notion might be in line with an earlier report that DBP regulates viral mRNA stability, suggesting that the DBP level is a critical determinant of infection progress (63). However, availability of the AdV DNA polymerase and TP would be equally essential. Our analysis showed that the initial recognizable RC structures grow autonomously in size, which is compatible with further addition of DBP oligomers but is soon followed by a much larger size increase through fusion to form early RC. We also confirmed our previous observation that early RC transform into late RC. Interestingly, while the onset of RC formation varied between cells and was dependent of the MOI, the relative kinetics of each step were similar between cells, suggesting a coordinated replication program. We also observed a strong correlation between the early-to-late transition of RC morphology with a slow-to-fast shift in genome amplification rates, which occurred concomitantly with the appearance of ViPR bodies as determined through the dynamic redistribution of tagged Mybbp1A. The direct labeling of replicated DNA allowed us to calculate apparent replication rates for both replication phases (10 versus 100 genomes/hour on average), showing a 10-fold difference in the genome replication rates between early and late replication. Calculated replication rates were based on the detection of individual *Anch* sequence-labeled vector genomes identifiable as OR3-GFP fluorescent spots after deconvolution-based image processing. These spots may contain more than one genome. In addition, they replicate only in the presence of limited amounts of untagged E1-positive genomes, which are also replicated, suggesting that we likely underestimate the number of replicated genomes. However, under our experimental conditions, all RC were OR3-GFP positive, and using two different MOIs resulted in similar calculated replication rates, suggesting that our quantification is a good approximation. At this point, it is unclear what triggers the kinetic transition in genome replication and the morphological changes in the RC. One possibility is that the expression level of DBP determines the morphology and functionality (i.e., replication rates) of RC, as suggested above. It is also possible that viral late genes encode a switching factor(s) for the early-to-late RC transition. As proposed previously (29, 32, 57), early RC could produce viral genomes as templates for late gene expression. As a result, a key virus-encoded late gene product(s) may reach a threshold level to ensure that viral late proteins are sufficiently expressed to start virion assembly and to massively produce viral genomes for packaging, resulting in RC transformation. This scenario would explain how AdV regulates viral genome functions, from late gene expression to genome packaging. Applying the ANCHOR3 system also confirmed our previous study showing that ViPR bodies enrich replicated AdV genomes and genome-associated factors, such as protein VII and TP, but are unlikely places of genome packaging and/or virion assembly (32). Our results are more consistent with genome packaging occurring at the peripheries of ViPR bodies, but details of AdV genome packaging require further investigation. A recent report using a conditional knockout system showed that AdV virions can be assembled efficiently even in the absence of protein VII (64). This suggests that protein VII deposition onto genomes is not a prerequisite for virion assembly, supporting a model where the deposition follows packaging of naked genomes into capsids. It remains possible, however, that the deposition takes place before genome packaging, which is more in line with the results in this and our previous studies (32). Regardless, the deposition of protein VII can be spatially and/or temporally uncoupled from genome packaging.

In summary, we established a direct imaging system for AdV genomes in living cells based on the ANCHOR3 technology. We established the versatility of this experimental system for real-time genome observation. We showed previously unknown differences in AdV replication kinetics between early and late phases of replication and demonstrated genome distribution between cells through chromatin anchoring. We believe that real-time imaging of AdV genomes through the ANCHOR3 system will be a great asset to help uncover the fate of viral or vector genomes and ill-defined steps during early and late phases of AdV infection, including genome packaging and virion maturation in living systems.

## MATERIALS AND METHODS

### Cells.

U2OS (ATCC HTB-96), H1299 (ATCC CRL-5803), and HEK293 (ATCC CRL-1573) cells were maintained in Dulbecco's modified Eagle's medium (DMEM)-Glutamax (Life Technologies) supplemented with 10% fetal calf serum (FCS). The transfection of plasmids was done using Lipofectamine 2000 (Life Technologies) according to the manufacturer's protocol.

### Viruses.

The ANCHOR3-tagged Ad5 vector (Ad5-ΔE1ΔE3-ANCHOR3-GFP) was generated as follows. Plasmid pSF-Ad5 containing a ΔE1/ΔE3 AdV type 5 genome and a multiple-cloning site in the E1 region was purchased (OG268; Oxford Genetics). Vectors were linearized using the ClaI restriction enzyme. Oligonucleotides containing a 15-nucloetide (nt) sequence identical to both sides of the linearized pSF-Ad5 (lowercase letters in primer sequence below) were used for PCR of the full autofluorescent cassette (uppercase letters in primer sequence below) containing the *ANCH*3 region and the OR3-GFP fusion under the control of the simian virus 40 (SV40) promoter (*ANCH*3_SV40_OR3_GFP_fwd, tcagctgacggcgatGAATTCCCATGTCAGCCGTTAAG, and *ANCH*3_SV40_OR3_GFP_rev, ggccgccccagcgatAGGACCCCGGTAACGAGG). Linearized pSF-Ad5 and the purified PCR product (NEB) were used in a Gibson assembly recombination reaction at different ratios, according to the manufacturer's recommendations. Recombinants were screened by restriction enzyme digestion. Positive clones were transfected in HEK293 cells to confirm vector detection by fluorescence microscopy and for autofluorescent virus recovery. A replication-deficient autofluorescent vector (Ad5-ΔE1ΔE3-ANCHOR3-GFP), a replication-deficient GFP-expressing Ad5 vector (Ad5-ΔE1ΔE3-GFP), and a replication-competent human Ad5-ΔE3 were amplified in complementing HEK293 cells and purified using double cesium chloride banding as described previously (44). Infection assays were performed at high MOI by adding 5,000 pp/cell (pp/c) of Ad5-derived vector to the medium for incoming-virus studies. For replication studies, the inoculum was supplemented at a ratio of 1:10 with 500 pp/c of replication-competent Ad5 to supply E1 expression. In some studies, the inoculum was diluted 1:10 in DMEM to lower the infection dose.

### Antibodies.

The primary antibodies used in this study were rabbit anti-lamin A/C, rabbit anti-pTP, rabbit anti-AdV particles, mouse anti-DBP, mouse anti-protein V, and rat anti-E4orf6 antibodies and were kind gifts provided by L. Gerace (TSRI, La Jolla, CA, USA), D. Matthews (University of Bristol, UK), R. Iggo (Institut Bergonie, Bordeaux, France), J. Flint (Princeton), and T. Dobner (HPI, Hamburg, Germany), respectively. Mouse anti-protein VII (41) and mouse anti-TAF-Iβ (65) have been described previously.

### Plasmids.

The expression vectors for OR3-GFP and OR3-NLS-GFP were provided by NeoVirTech. The expression vectors for mCherry-tagged USP7 and Mybbp1A (pcDNA3-mCherry) were obtained from Montpellier Genomic Collections (Institut de Génétique Moléculaire de Montpellier) (MGC). The expression vector for histone H2B-tdiRFP (pCAG-H2B-tdiRFP-IP) was obtained from M.-E. Torres-Padilla's laboratory (Université de Strasbourg) via Addgene (plasmid 47884) (66).

For the preparation of cells stably expressing mCherry-tagged USP7 and Mybbp1A and tandem dimeric near-infrared red fluorescent protein (tdiRFP)-tagged histone H2B, U2OS cells were transfected with the corresponding expression vector and cultured for 2 weeks in the presence of 2 mg/ml G418 or 2 μg/ml puromycin, respectively. Stable fluorescent cells were enriched to homogeneity by fluorescence-activated cell sorting. Cells stably expressing mCherry-tagged TAF-Iβ were described previously (44).

### Immunofluorescence analysis.

Indirect immunofluorescence (IF) analyses were carried out as described previously (44). Fixed and mounted IF samples were analyzed using a Leica SP5 confocal microscope. Confocal stacks were taken every 0.3 μm, and images were processed using ImageJ and presented as maximum-intensity projections.

### Live-cell imaging.

For live-cell imaging, cells were seeded in ibidi μ-Slide VI 0.4 (Ibidi), and images were acquired using either a Leica spinning-disk microscopy system (100× objective) equipped with an environmental chamber at 37°C; a Zeiss Axio Observer Z1, Apotome 2 wide-field fluorescence microscope with an environmental system; or an inverted Leica DM6000 wide-field microscope equipped with an environmental chamber and a CMOS (complementary metal oxide semiconductor) camera. The conditions of acquisition are detailed in the respective movie legends. The movies were assembled using ImageJ.

### FRAP analysis.

FRAP analysis of individual genomes was done with an SP5 Leica confocal microscope using the Leica FRAP mode. ANCHOR3 spots depicting individual genomes were preimaged for 3 frames at 3-s intervals, followed by bleaching at ∼75% laser power. The bleach power and area were optimized manually and restricted to the immediate surroundings of the signal. Postbleach recovery was imaged at 3-s intervals for ∼210 s. The signal intensities measured were background corrected against the same cell and normalized for comparison. The best fit and 95% CI for the half-recovery time from >40 genomes was calculated using PRISM7 software.

### Imaging analysis.

The image-processing pipeline for the dynamic quantification of viral genomes (OR3-GFP signal) and cellular marker (mCherry-USP7/mCherry-Mybbp1A) was as follows. For each experimental condition, a cell was selected, isolated, and tracked during the entire acquisition time. The channel depicting the viral genome (OR3-GFP signal), the RC (mCherry-USP7 signal), or ViPR bodies (mCherry-Mybbp1a) was then split into individual channels to process the two images independently, using the best processing parameters for each signal. To produce quantifiable signals, an image-processing scheme was developed. The background signal was first estimated and removed, and a deconvolution based on the alternating direction method of multipliers (ADMM) (67, 68) was then performed to reduce both noise and blur in the temporal series of images. The point spread function (PSF) used in the deconvolution process was estimated locally on the OR3-GFP signal channel, using an average of the smallest replication sites containing only one copy of the signal. The number, the size, and the intensity of individual objects of interest were then estimated on each channel after segmentation, computed frame by frame, and plotted over the acquisition time. To calculate genome replication rates, a linear least-squares regression was performed on two different phases of the OR3-GFP signal, namely, the early “slow” replication and late “fast” replication stages.

### SDS-PAGE and Coomassie gels.

SDS-PAGE was done using 11% polyacrylamide (40% acrylamide and bis-acrylamide solution; 37.5:1; Bio-Rad) gels, which were stained with Coomassie blue as described previously (9).

## Supplementary Material

Supplemental file 1

Supplemental file 2

Supplemental file 3

Supplemental file 4

Supplemental file 5

Supplemental file 6

Supplemental file 7

Supplemental file 8
