# Supplementary material for: In Vivo Labelling of Adenovirus DNA Identifies Chromatin Anchoring and Biphasic Genome Replication
Source: J Virol. 2018 Aug 29;92(18):e00795-18. doi: 10.1128/JVI.00795-18 (PMC6146703; doi:10.1128/JVI.00795-18)
Supplement: Supplemental file 8 [file zjv018183871s8.pdf]

Supplemental Material

Legends for supplemental Movie Files

Movie S1A-C: U2OS epithelial cells stably expressing mCherry-TAF-Iβ (magenta signal) were transiently transfected with OR3-GFP expression plasmid (green signal). Cells were mock-infected (Movie S1A), infected with Ad5-ΔE1ΔE3-GFP (Movie S1B) or infected with Ad5-ΔE1ΔE3-*Anch*OR3-GFP (Movie S1C). At ~3 hpi cells were imaged by spinning disk confocal microscopy at a single Z-section at 1 frame/3 sec interval. Raw data were assembled in ImageJ and saved at 10 frames/sec.

Movie S2: U2OS epithelial cells were transiently transfected with OR3-GFP expression plasmid (green signal). Cells were infected with Ad5-ΔE1ΔE3-*Anch*OR3-GFP and 5 Z-stacks at 0.3 μm were acquired every 8 min using a widefield microscope equipped with a CMOS EMCCD camera driven by Metamorph software. Movies were assembled using best focal plane selection and displayed at a frame rate of 5 frames/sec. The right channel depicts the unprocessed data and the left channel the same data following deconvolution using MetaMorph's nearest neighbor deconvolution analysis tool.

Movie S3: U2OS epithelial cells stably expressing fluorescent histone H2B (tdiRFP, red signal, right channel) were transiently transfected with OR3-GFP expression plasmid (green signal, left channel). Cells were infected with Ad5-ΔE1ΔE3-*Anch*OR3-GFP and at ~3 hpi screened for cells displaying condensed chromosomes. Cells with condensed chromosomes were than imaged at 1 frame/20 sec interval using a single Z-section on a spinning disk confocal microscope throughout cell division. Movies were assembled in ImageJ and displayed at a

frame rate of 3 frames/sec. The right channel depicts chromosomes, the left channel chromosome associated AdV genomes, and the middle panel a merge of both channels.

Movie S4: U2OS epithelial cells stably expressing mCherry-USP7 (red signal, left channel) were infected with an excess of Ad5-ΔE1ΔE3-*Anch*OR3-GFP in presence of replicative Ad5-ΔE3 and imaged at 1 frame/20 min interval using a 5 Z-section Zeiss Axio Observer Z1, Apotome 2 wide-field fluorescence microscope. Movies were assembled in ImageJ and Z-projections are displayed at a frame rate of 10 frames/sec starting at 12 hpi. The left channel depicts USP7 marking replication centers (RC), the right channel depicts replicated AdV genomes via the *Anch*-sequence-associated OR3-GFP signal and the middle panel is a merge of both channels.

Movie S5: U2OS epithelial cells stably expressing mCherry-Mybbp1A (red signal, left channel) were infected with an excess of Ad5-ΔE1ΔE3-*Anch*OR3-GFP in presence of replicative Ad5- ΔE3 and imaged at 1 frame/20 min interval using a 5 Z-section Zeiss Axio Observer Z1, Apotome 2 wide-field fluorescence microscope. Movies were assembled in ImageJ and Z-projections are displayed at a frame rate of 10 frames/sec starting at 9 hpi. The right channel depicts Mybbp1A, the left channel depicts replicated AdV genomes via the *Anch*-sequence-associated OR3-GFP signal and the middle panel is a merge of both channels.
